# Joint pain epidemiology and analgesic usage in Madagascar

**DOI:** 10.11604/pamj.2017.26.77.11215

**Published:** 2017-02-20

**Authors:** Luc Hervé Samison, Fidiniaina Mamy Randriatsarafara, Stéphane Ralandison

**Affiliations:** 1 University of Antananarivo, Hospital Joseph Ravaoahangy Andrianavalona, Antananarivo, Madagascar; 2 University of Antananarivo, Centre Hospitalier de Soavinandriana, Antananarivo, Madagascar; 3 University of Toamasina, Hospital Morafeno, Toamasina, Madagascar

**Keywords:** Arhtralgia, joint disease, analgesic, Africa, Madagascar

## Abstract

**Introduction:**

To describe the epidemiology of joint pains and document analgesics usage in an African context.

**Methods:**

Patients suffering from joint pain were recruited from nine sites located in Antananarivo, Madagascar, including 6 hospital services and 3 clinics. Doctors collected information on the etiology and characteristics of the patients’ pain. Analgesics prescribed by these doctors were also documented.

**Results:**

In total, 400 patients were enrolled in the study (52.5% women, mean age of 42.34 years ± 17.7 [4-86]). Pain of mechanical type was found in 260 participants, 65%; 95% CI [60.1% to 69.6%] and inflammatory type pains in 128 cases 32%; 95% CI [27.5% to 36.9%]. Mixed pains were found in 12 patients (3%). The median duration of pain prior to the consultation was 6.5 days. The average pain intensity was 57.9 ± 19.9 mm of a total of 100 mm maximum on a visual analogue scale, VAS. The etiologies of mechanical type pains were dominated by fracture, common low back pain and tendonitis. Arthrosis was the dominant cause of inflammatory type pain, followed by rheumatoid arthritis and gout. NSAIDs (74.5%) were the most frequently prescribed analgesics followed by paracetamol (49.5%), weak opioids (23%) and corticosteroids (12.25%). Two-thirds of medical prescriptions (65.3%) were of combined analgesics.

**Conclusion:**

These findings demonstrated that mechanical type pains were the main reason for consultations for joint pain in these situations in Antananarivo, Madagascar. The most frequently prescribed pain-relieving medications were NSAIDs, paracetamol, weak opioids and corticosteroids. This descriptive study may be a useful starting point for further epidemiological studies of pain in the African context.

## Introduction

Pain and its management remain a serious public health problem worldwide. Pain represents the main reason for consultation (50% of medical consultations). The costs of its management vary from one country to another. In the United Kingdom, back pain management costs to society were found to be £6.600 million to £12.300 million [1]. Pain is often disabling and can reduce the quality of life [2]. Several guides giving the characteristics of pains of different etiology and their management have been published [3]. The exact formulation of various drugs including analgesics may vary somewhat from country to country as may the drug most commonly used in a particular disease depending on medicine availability and on the local public health context.

In the African world, research on the use of analgesic on joint pain is almost non-existent. Indeed, there are very few publications that deal with this subject. With the keyword « joint pain, analgesic, Africa », only 17 publications were found from the PubMed database in August 2015 of which two directly related to the assessment of the effects of analgesic in arthritis [4, 5] and about half of them are related to viral infections. This result prompted us to conduct a study in which the main objective was to describe the characteristics of patients with joint pains treated with analgesics in an African context where prescription is based on the purchasing power of patients. The secondary objectives were to assess the pain intensity at enrollment, to list the diseases involved, and finally to analyze the analgesic prescription.

## Methods

### Participants and protocol

The study was a multicenter descriptive, cross sectional, observational and quantitative study conducted during routine medical consultations in Antananarivo, capital of Madagascar. Four hundred patients were recruited in 3 months from April 2^nd^ 2013 to July 2^nd^ 2013 from nine different sites (6 hospital outpatient services and 3 clinics, with each centre providing from 10 to 150 cases/participants). Outpatients coming to a private clinic or to an emergency service or any recent inpatients coming to hospital for joint nociceptive pain regardless of duration with either acute or chronic pain were included. Patients with bone or joint pain following surgery, or due to cancer, and those with neuropathic pain or other types of pain were excluded.

Nine doctors on the nine recruitment sites led the study. On each site, two or three other physicians were assigned the inclusion of patients with joint pain. Variables entered onto the proforma used in this study included personal information, clinical parameters such as characteristics of pain, diagnoses and the analgesics prescribed by physicians. For each patient, information was recorded on survey sheets after obtaining their informed signed consent.

### Statistical analysis

Data were entered and analyzed using Epi-info software 3.5.3. The Site-Patient codes were the unique ID for each patient. Each file had an individual assigned code. After entering the 400 cases, missing data on mandatory fields and some outliers was controlled and verified on each sheet. Related corrections was done first on Epi Info and then on Excel 2007. Quality control of data entry was undertaken by random sampling and checking 10% of the data entered. The texts, graphics, and tables were produced using Excel 2007 and Word 1999-2003 and Word 2007 software. Qualitative variables were expressed as proportions presented as percentages and graphically; quantitative variables were expressed as arithmetic means. A 95% confidence interval was given for many results these being given as means + standard deviation with medians being given with minimum and maximum values.

For comparisons, the selected significance threshold chosen corresponded to p < 0.05. Statistical analysis was made in univariate. For comparisons of percentages Chi-square was used with corrections using the Fischer test or the test of Yates for low numbers. To give acceptable statistical power, for a total population of 22,000,000 in Madagascar, a representative sample of 385 patients was sufficient. Calculation of the sample size was made in a rational way as the ability of the center to recruit new patients.

### Ethics

Clinical data confidentiality and the anonymity of the patients have been followed throughout the process of inclusion and analysis.

## Results

Recruitment objectives were achieved at 100% (R/0=100%) with the recruitment of 400 patients during the study period. One hundred and ninety patients were been enrolled in 4 emergency units, 150 were recruited in a Rheumatology service, 45 in three different private clinics and 15 in a trauma surgery service.

### Subject characteristics

One hundred and ninety men and 210 women were recruited with a sex ratio of 0.9. Their mean age was 42.34 ± 17.7 years. This distribution was symmetrical with a median at 42 years, a minimum of 4 years and a maximum of 86 years. Full-time workers represented 40.3% of the sample and the unemployed represented one fifth (19.3%).

### Pain characteristics

Physicians stratified the patients on presentation by diagnosis using a pre-established list of diseases; secondary classification was done at the data analysis step. The complaints of the patients were classified in terms of the type of pain with of mechanical pain occurring in 260 cases (65.0 %; 95% CI 60.1% to 69.6%) and inflammatory type of pain presenting in 128 cases (32.0%; 95% CI 27.5% to 36.9%). Twelve patients had joint pain combining features of both types of pain.

**Figure 1:**
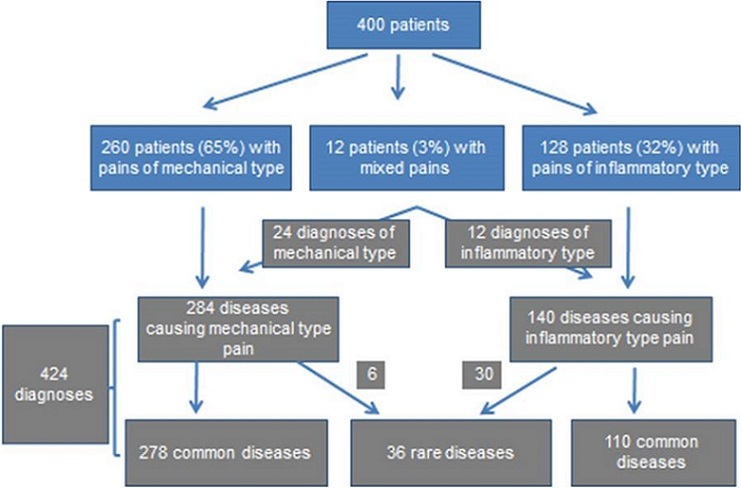
Distribution of patients and diagnoses evoked by physicians

Diseases causing joint pain were divided in two groups, those where there is a mechanical type pain and those which have an inflammatory type pain. Twenty four patients had two diagnoses which caused joint pains. Thirty-six rare diseases have been identified including joint sickle cell crisis, chondropathy, hallux valgus, scoliosis and articular manifestations of psoriasis as seen in Figure 1.

Frequencies of pain types depending on patient ages were shown in Table 1 as is the frequency of inflammatory pain which increased with age.

**Table 1:** Distribution of pain types and ages (n=400)

Ages	Types of pain							p
	Mechanical			Inflammatory		Mixed		
	N	n	%	n	%	n	%	
19 years and under	43	35	81.4	6	14.0	2	4.7	0.004
20 to 39 years	127	94	74.0	30	23.6	3	2.4	
40 to 59 years	163	94	57.7	63	38.7	6	3.7	
60 years and over	67	37	55.2	29	43.3	1	1.5	
TOTAL	400	260	65.0	128	32.0	12	3.0	

Osteoarthritis (40.9%) was the most frequent disease causing inflammatory pain type, followed by rheumatoid arthritis (20%), gout (14.5%), undetermined arthritis (12.7%), connective diseases (5.5%) and other rare diagnoses. Osteoarthritis was here classified in inflammatory diseases because flares-up had caused inflammatory type pain at the time of consultation even though there was a mechanical basis of the chronic pain. A positive correlation was found between body mass index and pain of inflammatory type as shown in Table 2.

**Table 2:** Distribution of types of pain and Body Mass Index BMI

BMI	Types of pain						p
	Mechanical		Inflammatory		Mixed		
	n	%	n	%	n	%	
BMI < 25 n=264	185	70.1	70	26.5	9	3.4	0.004
Overweight n=111	65	58.6	43	38.7	3	2.7	
Obese n= 25	10	40.0	15	60.0	0	0.0	
TOTAL	260	65.0	128	32.0	12	3.0	

Where mechanical type pain was found, the three most common diagnoses were articular fractures (24.5%), nonspecific knee pain (17.4%) and common low back pain (15.8 %) followed by tendonitis (14.7%), sprain (11.5%), dislocation (9%), common back pain (4.3%) and common neck pain (2.9%).

### Magnitude and duration of pain

Mean pain magnitude assessed with Visual Analogue Scale (0-100) was 57.9 ± 19.9 mm with a median of 60 the range being from 10 to 100. Moderate pain (30 to 60 mm) was found in three quarters of patients painful events (75.5%). Mild pain was present in 4.8% and severe pain in 19.8%. Mean pain duration was 265.30 ± 797.94 days, with a median of 6.5 days, ranging from 1 day to 5475 days or 15 years. Acute pain of less than 3 weeks was present in almost two thirds of patients (61.5%). Patients who had subacute pain constituted 16% of the whole and chronic pain with duration more than three months represented 22.5%.

### Analgesics

Considering on the various categories of analgesics, non-steroidal anti-inflammatory drugs (NSAIDs) were the most prescribed (74.5%), followed by analgesics level 1 (49.5%), analgesic level 2 (23%) and corticosteroids (12.25%) as listed in Table 3. These four analgesic categories represented 88.11% of all prescriptions.

**Table 3:** Analgesic categories prescribed during enrollment. Total number of analgesics (n=723)

Analgesic categories	n	% on 400
NSAID	298	74.50
Analgesic level 1 (paracetamol)	198	49.50
Analgesic level 2 (weak opioid)	92	23.00
Corticosteroid	49	12.25
Antimitotic	19	4.75
Anti-gout	16	4.00
Major opioid	14	3.50
Muscle relaxant	14	3.50
Others	23	5.75

Two-thirds of prescriptions (65.3%) were of tablets in which two analgesics were combined. The analgesics prescribed, with their dosage forms have been listed in order of frequency. The most prescribed was paracetamol in tablet form (38.25%) followed by ketoprofen gel and the combined paracetamol + codein tablet as listed in Table 4.

**Table 4:** Analgesics and dosage forms prescribed during enrollment (n=723)

Analgesic prescribed and dosage forms (INN)	N	% on 400
Paracetamol tablet	153	38.25
Ketoprofen gel	57	14.25
Paracetamol + Codeine tablet	57	14.25
Ketoprofen suppository	50	12.50
Ketoprofen tablet	45	11.25
Diclofenac tablet	35	8.75
Diclofenac gel	33	8.25
Prednisone tablet	28	7.00
Tramadol tablet	23	5.75
Colchicine tablet	16	4.00
Diclofenac capsule	16	4.00
Injectable Morphine	15	3.75
Other analgesics (<15 prescriptions)	195	48.75

**Figure 2:**
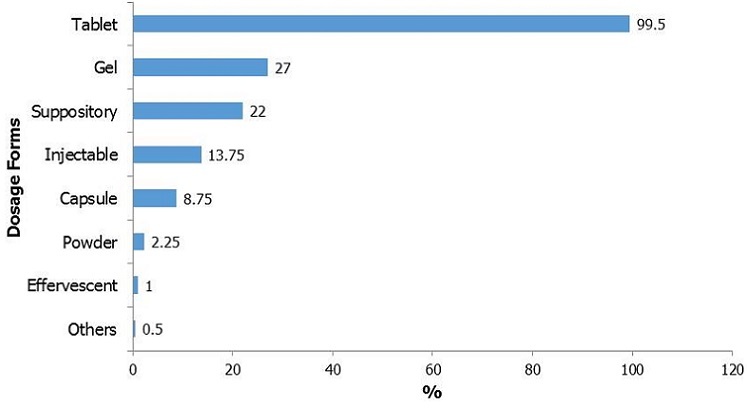
Distribution of dosage forms (n=723), percentage on 400 patients

Tablets were prescribed in almost all patients, followed by topical gels and suppositories as shown in Figure 2. More than half of the patients in the study (55.7%) had already had analgesic drug therapies prior to the consultation.

## Discussion

Analgesics are widely used in the management of joint pains. In our study fracture was found to be the main cause of mechanical pain type and degenerative joint disease was main cause of inflammatory pain type entailing analgesics prescription. The most prescribed analgesic drug for these diseases, namely the paracetamol, was used alone or in combination with an NSAID.

Our study population study was relatively young, with an average age of 42.34 years. There was a clear division between mechanical type pains that predominated in young people and inflammatory type pains which predominated in the elderly. Here there was a high incidence of osteoarthritis in those over 45 years, and who were frequently seen during an inflammatory exacerbation of their disease. This finding in our study is corroborated by other studies confirming that the coxarthrosis, gonarthrosis and low back pain are the main reasons for consultations for osteoarticular diseases [6, 7]. In addition, the positive correlation between BMI and inflammatory type pain found in our study supports the known association between age, excess weight and osteoarthritis noted elsewhere [8]. Conversely, mechanical pain was much more frequent among young people in whom there were a large number of traumatic diseases (fractures) and micro-traumatic diseases (tendonitis and sprains).

In our study, the major diseases causing inflammatory type pain were osteoarthritis, rheumatoid arthritis and gout. Although osteoarthritis is a degenerative condition usually manifested by mechanical type pain, patients in our series only seemed to consult when there was an inflammatory flare up. It is known that the delay to diagnosis is long in Madagascar and Africa perhaps because of the cost of treatment which is mostly supported by the patients [9]. In rheumatoid arthritis, the diagnostic delay is 43 months according to a study in Antananarivo [10]. In microcrystalline intrasynovial deposit arthritis, in the absence of polarized light microscopes, formal identification of the type of crystals and hence diagnosis is difficult. Diagnosis is therefore mainly clinical before the occurrence of stereotypical crises, when it normally is based on the inflammatory nature of the joint fluid.

The intensity of pain in this study averaged 57.9 ± 19.9 mm for an average duration of 265.30 days. This intensity is high compared with other studies but may be associated with the predominance of traumatic pain in our study where the pain is acute. The average was lower in chronic pain, being 44 + 23 mm for an average duration of 7.4 years [11]. In a decreasing frequency order, the five most prescribed analgesics in our study were: paracetamol in tablet form, paracetamol + codein in association in tablets and ketoprofen in its suppository, tablet or gel dosage forms. The first four most prescribed therapeutic categories were: NSAIDs, analgesics, weak opioids, and corticosteroids. Prescribers in our study tended to use NSAIDs and weak opioids in young patients; paracetamol was used in all groups regardless of age. British general practitioners prioritize paracetamol for joint pain whatever the joint involved. This prioritization accounted for 84% of prescriptions, followed by 70% of opioid [12]. The use of paracetamol is particularly favoured for elderly patients as it is effective for both acute pain and chronic pain [7]. NSAIDs should be used with care in elderly persons bearing in mind their adverse effects and they should be prescribed at the minimum effective dose and for a short duration [7]. Several authors suggest using coated NSAIDs tablets and prescribing proton pump inhibitors to reduce their adverse effects [13–15].

As pain was only moderate in many patients in our study, the prescription of weak opioids is less than might be expected because of the effectiveness of NSAIDs. This choice of the NSAID is commonly seen in young populations especially in the pediatric world where their effectiveness and safety are widely appreciated [16]. NSAIDs also have an advantage for non-specific lower back pain as they both lessen the pain and facilitate movements whereas other therapeutic categories have only analgesic actions [17]. The use of weak opioids has been studied in rheumatoid arthritis and they are rarely recommended because of their frequent adverse effects which can diminish the benefits of this category of drugs, particularly among the elderly [18].

Muscle relaxants were rarely prescribed in our study. According to a published review, in patients with rheumatoid arthritis, they bring no demonstrated benefit after one week. Adverse effects such as drowsiness and dizziness are significantly elevated even with short duration administration [19].

Injection of corticosteroids provides a short term relief (four to eight weeks) of the flare-ups of osteoarthritis of the knee while visco-supplements by hyaluronic acid can maintain the improvement of symptoms for longer period [20]. The low uptake of these products in developing countries is due in part by the limitation injections as they are only done by specialists, and the lack of product access to visco-supplements because of their expense. Among the 400 patients, 55.7% had already taken analgesic before their entry in the study, mostly as self-medication. This is confirmed by other study. In a German study, the most frequently analgesic taken were NSAIDs (45.6%), NSAIDs and Paracetamol in association with other drugs (42.6%) and NSAIDs in association with another analgesic [21].

One limitation of our study is that recruitment came from limited sources predominantly hospital consultations, hospital emergencies departments and recent inpatients in rheumatology service. Thus there is a strong bias to the hospital. It would be useful to replicate the study in remote health centers and private clinics in towns to determine the characteristics and causes of such pain of the majority of the population. First contacts of patients with the Malagasy health system are made in these centers. Consultations at general practitioners may give similar findings.

## Conclusion

Mechanical type pains were the main reasons for consultations for joint pain in Antananarivo. The etiologies of these pains were dominated by fracture, common low back pain and tendonitis. The main causes of inflammatory pain were flare-ups of osteoarthritis, rheumatoid arthritis and gout. A positive correlation was demonstrated between advanced/advancing age and pain of inflammatory type and in the young mechanical type pain predominated. Two-thirds of patients consulted for acute pain. The average pain intensity was moderate. The most frequently prescribed analgesic were NSAIDs, paracetamol, weak opioids and corticosteroids. It may be useful to do similar studies of joint pains in different situations in Africa to determine their causes and the frequency of various methods of pain management.

### What is known about this topic

- Joint pain type in developed country;
- Analgesics use in developed country.

### What this study adds

- Types of joint pain in a developing country (Madagascar);
- Joint pain characteristics;
- Main analgesics used to treat join pain in a developing country (Madagascar).
